# Meta-DiSc 2.0: a web application for meta-analysis of diagnostic test accuracy data

**DOI:** 10.1186/s12874-022-01788-2

**Published:** 2022-11-28

**Authors:** Maria N. Plana, Ingrid Arevalo-Rodriguez, Silvia Fernández-García, Javier Soto, Martin Fabregate, Teresa Pérez, Marta Roqué, Javier Zamora

**Affiliations:** 1 Health Technology Assessment Unit, Hospital Universitario Ramón Y Cajal, IRYCIS, Madrid, Spain; 2 CIBER of Epidemiology and Public Health, Madrid, Spain; 3 Clinical Biostatistics Unit, Hospital Universitario Ramón Y Cajal, IRYCIS, Madrid, Spain; 4 Radiology Department, Hospital Universitario Ramón Y Cajal, IRYCIS, UPM, Madrid, Spain; 5 Internal Medicine Department, Hospital Universitario Ramón Y Cajal, IRYCIS, Madrid, Spain; 6 Department of Statistics and Data Science, Complutense University of Madrid, Madrid, Spain; 7 Barts Research Centre for Women’s Health, WHO Collaborating Centre, Queen Mary University of London, London, UK; 8 Iberoamerican Cochrane Center, Biomedical Research Institute Sant Pau (IIB Sant Pau), Barcelona, Spain; 9 WHO Collaborating Centre for Global Women’s Health, Institute of Metabolism and Systems Research, Institute of Applied Health Research, University of Birmingham, Birmingham, UK

**Keywords:** Systematic review, Meta-analysis, Sensitivity, Specificity, Diagnosis, Heterogeneity

## Abstract

**Background:**

Diagnostic evidence of the accuracy of a test for identifying a target condition of interest can be estimated using systematic approaches following standardized methodologies. Statistical methods for the meta-analysis of diagnostic test accuracy (DTA) studies are relatively complex, presenting a challenge for reviewers without extensive statistical expertise. In 2006, we developed Meta-DiSc, a free user-friendly software to perform test accuracy meta-analysis. This statistical program is now widely used for performing DTA meta-analyses. We aimed to build a new version of the Meta-DiSc software to include statistical methods based on hierarchical models and an enhanced web-based interface to improve user experience.

**Results:**

In this article, we present the updated version, Meta-DiSc 2.0, a web-based application developed using the R Shiny package. This new version implements recommended state-of-the-art statistical models to overcome the limitations of the statistical approaches included in the previous version. Meta-DiSc 2.0 performs statistical analyses of DTA reviews using a bivariate random effects model. The application offers a thorough analysis of heterogeneity, calculating logit variance estimates of sensitivity and specificity, the bivariate I-squared, the area of the 95% prediction ellipse, and the median odds ratios for sensitivity and specificity, and facilitating subgroup and meta-regression analyses. Furthermore, univariate random effects models can be applied to meta-analyses with few studies or with non-convergent bivariate models.

The application interface has an intuitive design set out in four main menus: file upload; graphical description (forest and ROC plane plots); meta-analysis (pooling of sensitivity and specificity, estimation of likelihood ratios and diagnostic odds ratio, sROC curve); and summary of findings (impact of test through downstream consequences in a hypothetical population with a given prevalence).

All computational algorithms have been validated in several real datasets by comparing results obtained with STATA/SAS and MetaDTA packages.

**Conclusion:**

We have developed and validated an updated version of the Meta-DiSc software that is more accessible and statistically sound. The web application is freely available at www.metadisc.es.

## Background

The evaluation of the role and properties of diagnostic tools has become a priority for global health policy and decision-making, driven mainly by the development of new technologies for well-known diseases and the emergence of new deleterious conditions affecting large-scale populations [1, 2]. Diagnostic evidence of the accuracy of a test for detecting a target condition of interest can be appraised using systematic approaches following standardized methodologies [3]. Briefly, diagnostic studies focus on estimating the ability of the index tool to identify subjects with or without the condition of interest [3]; evidence synthesis then requires two quantities: test sensitivity and specificity and the correlation between them [4]. The statistical approach used depends on the choice between estimating accuracy for a common threshold (i.e. an average operating point), or an expected curve across many thresholds (i.e. a summary ROC curve) [5–7], using commercial software packages with the analytical characteristics needed for fitting complex hierarchical models.

We recently found that the statistical synthesis of accuracy data was one of the methods more frequently omitted during the development of rapid reviews of diagnostic tests [8]. This, then, would be a potential bottleneck for the extended evaluation of diagnostic tools [8]. For several years, Meta-DiSc software has been one of the most widely used statistical programs in the meta-analysis of diagnostic data, with more than 1300 citations in peer-reviewed scientific articles [9]. It is a freely available, easy-to-use tool, that enables reviewers to apply statistical methods for the meta-analysis of diagnostic test accuracy (DTA) within an evidence synthesis framework. This software implemented the statistical methods recommended during its development, including the linear model proposed by Littenberg and Moses, and the univariate I-squared index to quantify heterogeneity. Hierarchical models are currently the method of choice for overcoming the limitations of previous statistical approaches [3]. These methodological developments have prompted us to update Meta-DiSc to include current statistical methods for the meta-analysis of test accuracy systematic reviews and an enhanced web-based interface to improve user experience. Our objective was to develop a new version of the Meta-DiSc software as a web application (app) to summarize DTA results by applying statistical methods based on hierarchical models.

### Implementation

We have developed a web-based app using R Shiny software. Shiny can be used to build R-based interactive applications directly on RStudio, the integrated development environment for R. The application has been deployed using the shinyapps.io platform.

#### Estimating pooled diagnostic accuracy indices

The app performs statistical analysis of DTA reviews using a bivariate random effects model [5] and the *glmer* function of the *lme4* package [10] for fitting a generalized linear mixed effect model. Summary points (average sensitivity and specificity) and the parameters are derived to depict the sROC curve. Positive and negative likelihood ratios (LR) and the diagnostic odds ratio (DOR) estimates are obtained from model parameters. The Delta method as implemented in the *msm* package [11] is used to compute the standard error of the estimates parameters. Forest plots and ROC plots have been implemented using the functionalities of the *meta*, *ggplot2* and *plotly* packages [12–14].

The program also offers the possibility of using a univariate random effects model. Although separate pooling is not recommended for DTA meta-analysis since it fails to account for the correlation between sensitivity and specificity, we have included this option because univariate models, in some instances, have a role in DTA reviews. This is the case, for example, when it is difficult to estimate all parameters of a bivariate model or when the focus of the analysis is only on one of the accuracy indices (*i.e*. sensitivity or specificity) [15].

#### Quantifying heterogeneity

Meta-DiSc 2.0 implements a thorough analysis of heterogeneity. In addition to the estimates of logit variances of sensitivity and specificity [16], the software calculates a bivariate I-squared index [17], the area of the 95% prediction ellipse using the *polyarea* function of the *pracma* package [18], and finally, the median odds ratios for sensitivity and specificity [16].

#### Exploring heterogeneity: subgroup and meta-regression analyses

The app can be used to perform subgroup and meta-regression analysis. For this purpose, additional columns need to be included in the dataset to define dichotomous covariates (one each time), which will be used to split the dataset and obtain the accuracy estimates for each subgroup. Exploring these individual results gives the reviewer insights into the between-group difference in sensitivity and specificity and the between-study variances in both indices. The meta-regression option compares the accuracy estimates obtained for these subgroups (i.e., sensitivity and specificity) [19]. The bivariate model includes interaction terms with both sensitivity and specificity and compares the statistical significance of these effects using the *lmtest* package [20]. For simplicity, meta-regression analysis implemented in Meta-DiSc 2.0 assumes that between-study variances are equal. Therefore, authors should check how appropriate is this assumption by comparing the between study variances in each subgroup.

## Results

Meta-DiSc 2.0 is freely available from www.metadisc.es. The user interface design is intuitive and easy-to-use. The left lateral panel organizes the workspace in four main menus: *File upload, Graphical description, Meta-analysis, and Summary of findings*. The app also includes a short user-guide video to show the practical use of the application.

### File upload menu

**Fig. 1:**
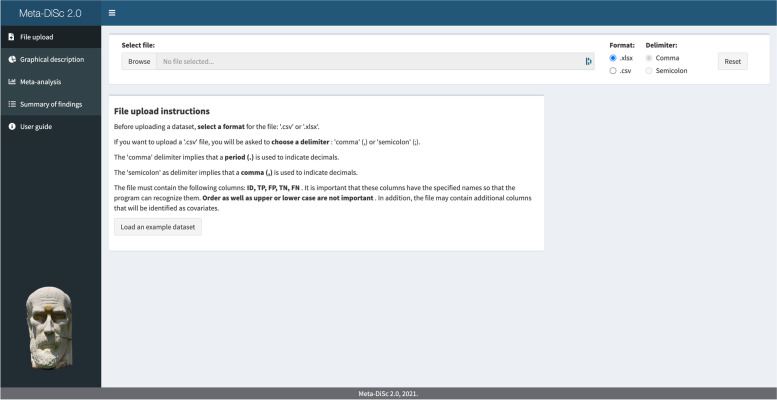
File upload menu

**Fig. 2:**
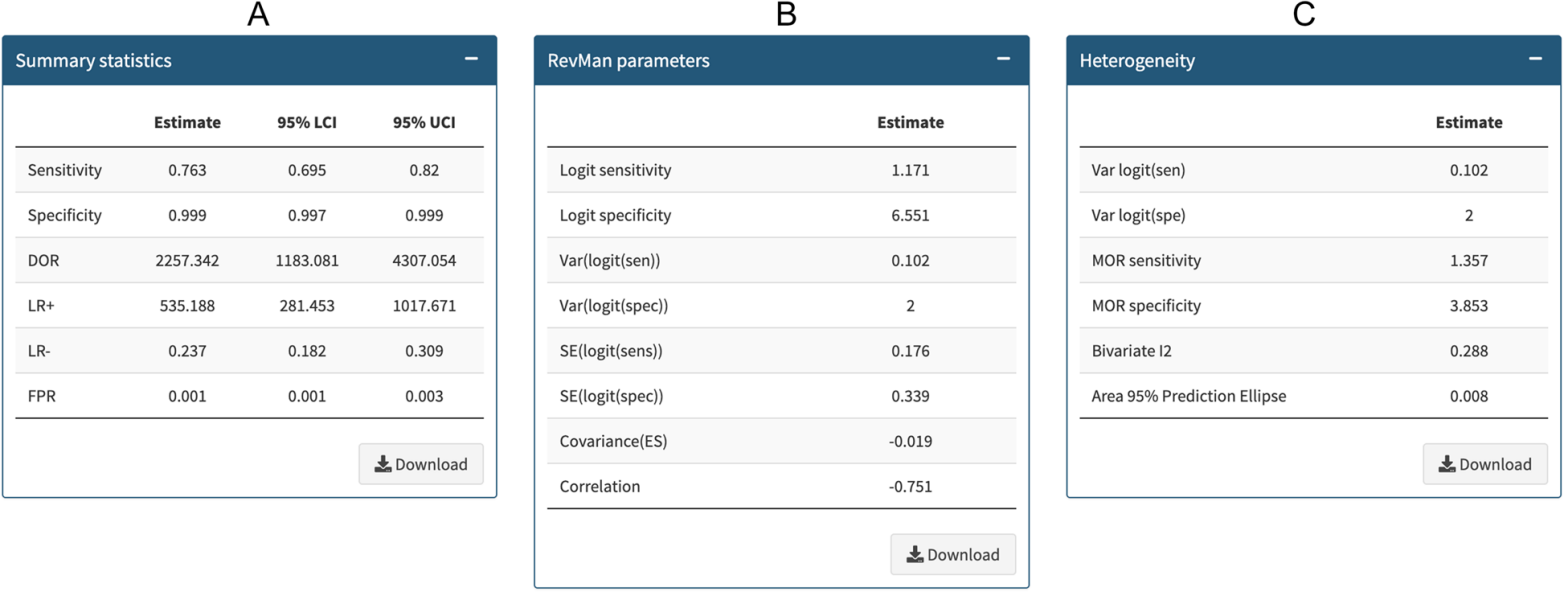
Bivariate analysis including summary statistics (**A**), model coefficients (**B**) and heterogeneity measures (**C**) (Meta-analysis menu)

**Fig. 3:**
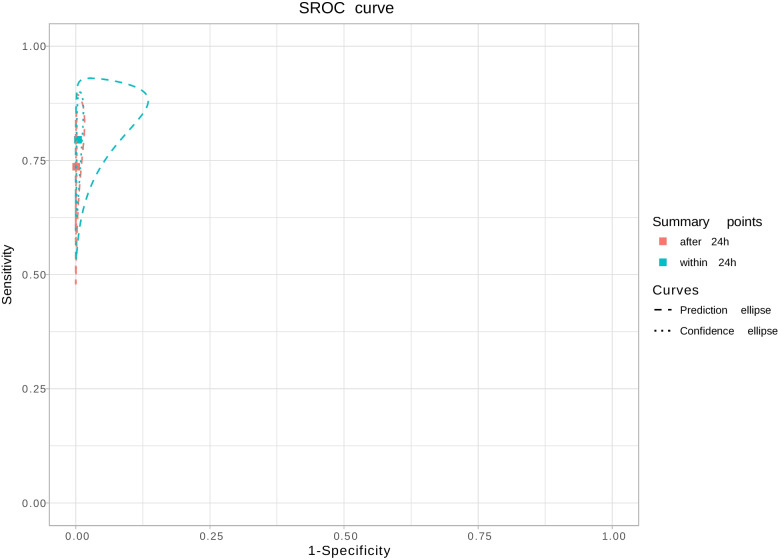
SROC curves in subgroup analysis (Meta-analysis menu)

**Fig. 4:**
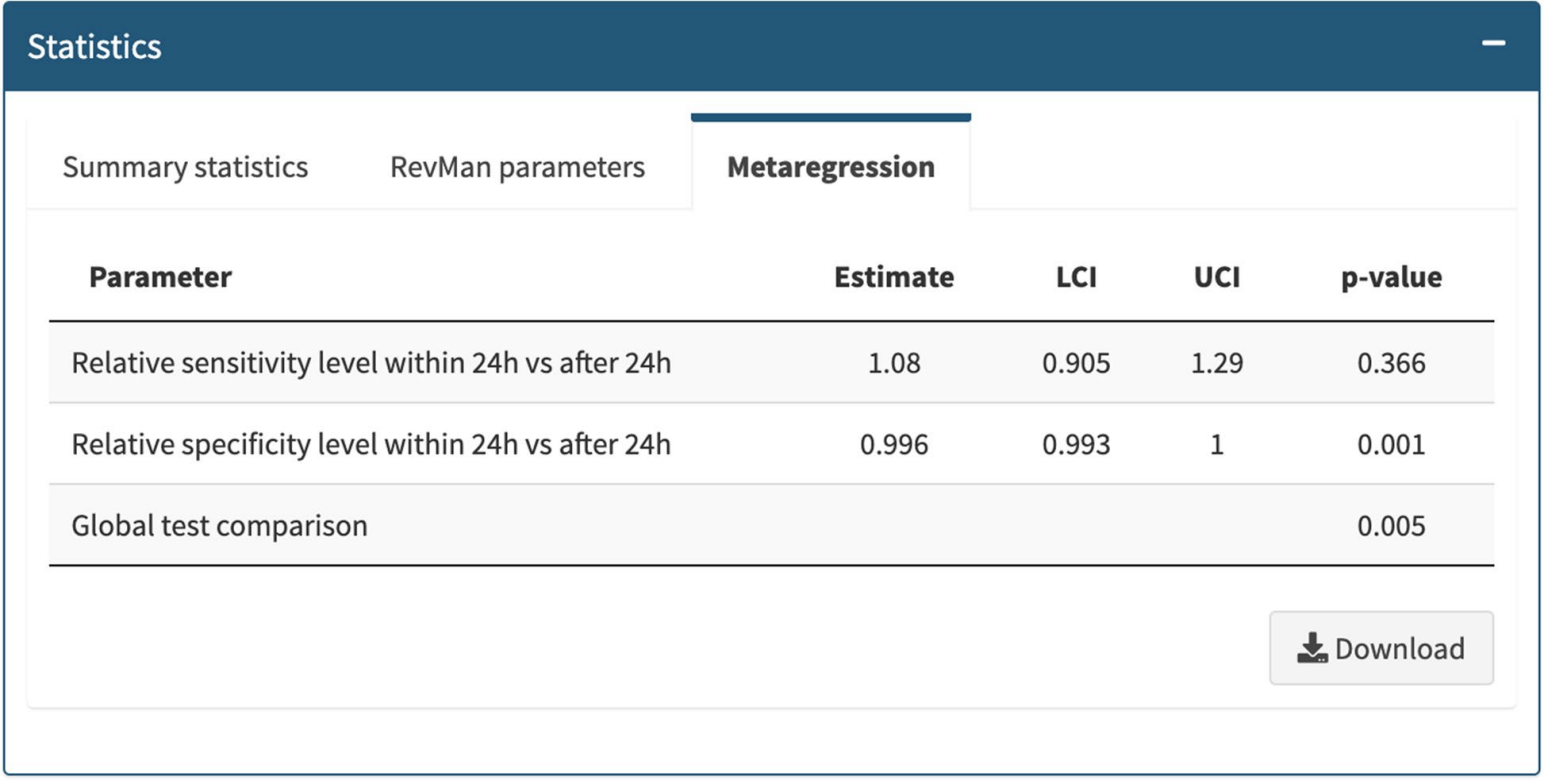
Metarregresion results (Meta-analysis menu)

**Fig. 5:**
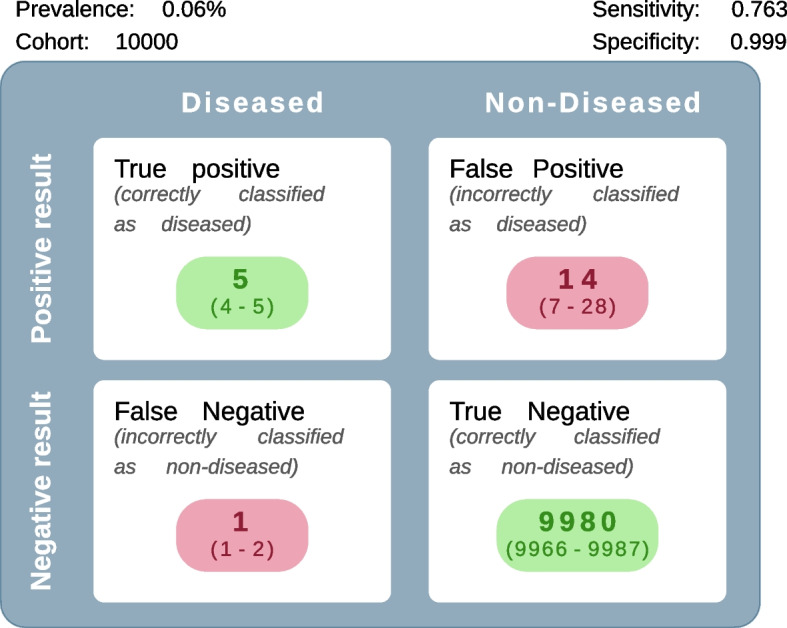
Summary of findings menu

The app can import data as either comma-delimited (i.e.,.csv) or Excel files (.xlsx files). The file must include data from 2 × 2 tables of individual studies in four columns named TP, FP, FN, TN, representing the number of true positives, false positives, false negatives and true negatives, respectively. The file must also include a unique identifier for each study (ID). It may also incorporate additional columns that will be considered as covariates to explore sources of between-study variability (Fig. 1). Figs. 2, 3, 4 and 5 show different app screens for the analysis of a published diagnostic accuracy systematic review on pulse oximetry screening for a critical congenital heart defects dataset [21].

### Graphical description menu

The app generates forest plots of sensitivity and specificity of individual studies to evaluate heterogeneity graphically. Studies of the forest plots are ordered in the same way as defined in the uploaded file. The ROC plot represents individual sensitivity and specificity, and offers the option of adding error bars, as either horizontal or vertical lines. This graphical description can be presented by subgroups defined by the covariates included in the file. All figures are downloadable as.png and.svg formats.

### Meta-analysis menu

All analyses are obtained from the meta-analysis menu. The first option of this menu is to fit the bivariate model of sensitivity and specificity. Results are shown in the corresponding tabs: i) statistics, ii) sROC curve, iii) subgroup analysis, and iv) sensitivity analysis.

In the statistics tab, users will find the pooled accuracy estimates (sensitivity and specificity, positive and negative likelihood ratios, diagnostic odds ratio and false-positive rate) along with their corresponding 95% CI (Fig. 2A). Additionally, the app provides model parameters estimates (logit sensitivity, logit specificity, standard errors, logits variances, covariance and correlation), which can be easily transferred to the Cochrane Review manager system (RevMan [22]) (Fig. 2B). Finally, the app shows the heterogeneity statistics, including variances of the logit sensitivity and specificity along with corresponding median odds ratios (MOR) [16], bivariate I-squared [17], and the area of 95% prediction ellipse [16] (Fig. 2C).

After visualizing these numerical results, users can obtain graphical summary results by moving to the next tab named sROC curve (Fig. 3), where the ROC plane graphic can be visualized and downloaded. Different display options can be selected or omitted, e.g., summary point, confidence and prediction ellipses, summary ROC curve, and individual study results.

The subgroup analysis tab fits a new bivariate model, including additional parameters to assess whether sensitivity and specificity differ between subgroups. After showing the coefficients of the estimated model, a formal comparison between subgroups can be made using the meta-regression tab. The app shows the relative sensitivity and specificity along with 95% confidence intervals (LCI and UCI) and p-values of likelihood ratio tests to compare the subgroups formed according to the selected covariate (Fig. 4).

A final sensitivity analysis tab can be used to restrict the analysis to certain specific studies, by simply selecting the level of the dummy variable that will be employed as the inclusion criterion from the dropdown menu.

If two independent univariate analyses of sensitivity and specificity are selected, the results of both random effects models are displayed in a series of screens showing pooled estimates, heterogeneity statistics, and forest plots.

### Summary of findings menu

To describe the absolute impact of a diagnostic test in a population with a given prevalence and fix a hypothetical sample size, the app calculates the number of false-positive and false-negative test results observed [23]. Users can download a figure that shows the outcomes (TP, FP, FN, TN) obtained (Fig. 5).

As a worked example, we have used a dataset that corresponds to a systematic review that evaluates the diagnostic accuracy of pulse oximetry as a screening method for detecting critical congenital heart defects (CCHD) in asymptomatic newborn infants [21]. The published meta-analysis included nineteen studies and was performed using the METADAS macro for SAS that uses Proc NLMIXED [24]. To assess potential sources of heterogeneity, we performed subgroup analyses and meta‐regression. The overall sensitivity of pulse oximetry for the detection of CCHD was 76.3% (95% CI 69.5 to 82.0), while specificity was 99.9% (95% CI 99.7 to 99.9). We measured total between-study variability in sensitivity and specificity through variances of the random effects for logit(sensitivity) and logit(specificity), and their covariance. We also provided 95% confidence and prediction ellipses.

We replicated the published analysis using Meta-DiSc 2.0, extending the heterogeneity description to include the area of the 95% prediction ellipse [16], the median odds ratio for sensitivity and specificity [16] and I2 bivariate [17] (Fig. 2C). We also replicated the subgroup analysis for the covariate "test of timing" (within 24 h of birth vs after 24 h from birth) (Fig. 3). Summary estimates of sensitivity and specificity of studies that performed screening after 24 h were 73.6% (95% CI 62.8 to 82.1) and 99.9% (95% CI 99.9 to 100). For studies that performed screening within 24 h, summary estimates of sensitivity and specificity were 79.5% (95% CI 70.0 to 86.6) and 99.6% (95%CI 99.1 to 99.8). The relative specificity for the detection of CCHD was significantly higher when newborn pulse oximetry was performed more than 24 h after birth (Fig. 4). Validation of the analyses using Meta-DiSc 2.0 produced the same results as those obtained with METADAS macro [24]. The comparison of the numerical results obtained with Meta-DiSc 2.0 and the results obtained with other software (METADAS in SAS [24], METANDI in Stata [25] and MetaDTA [26]) are shown in Table 1. We have further evaluated the app, replicating the analysis of four systematic reviews published in the literature [27–30] (Table 1).

**Table 1:** Metanalysis results using Meta-DiSc 2.0, METANDI (STATA), METADAS (SAS) and MetaDTA statistical software

First author, year	Software	Sensitivity (95% CI)	Specificity (95% CI)	LR + (95% CI)	LR- (95% CI)	DOR (95% CI)
Fahey et al. 1995 [27]	Meta-DiSc 2.0	0.66 (0.58; 0.73)	0.75 (0.68; 0.80)	2.58 (2.14; 3.11)	0.46 (0.39; 0.55)	5.59 (4.34; 7.19)
	METANDI	0.66 (0.58; 0.72)	0.75 (0.68; 0.80)	2.58 (2.14; 3.12)	0.46 (0.39; 0.55)	5.58 (4.33; 7.20)
	METADAS	0.66 (0.58; 0.72)	0.75 (0.68; 0.80)	2.58 (2.14; 3.12)	0.46 (0.39; 0.54)	5.59 (4.34; 7.20)
	MetaDTA	0.66 (0.58; 0.73)	0.75 (0.68; 0.80)	2.58 (2.14; 3.11)	0.46 (0.39; 0.55)	5.59 (4.34; 7.19)
Scheidler et al. 1997 [29]	Meta-DiSc 2.0	0.67 (0.61; 0.74)	0.84 (0.76; 0.89)	4.14 (2.70; 6.36)	0.39 (0.31; 0.49)	10.64 (5.83; 19.40)
	METANDI	0.67 (0.60; 0.74)	0.84 (0.76; 0.89)	4.15 (2.69; 6.40)	0.39 (0.31; 0.49)	10.65 (5.81; 19.54)
	METADAS	0.67 (0.60; 0.74)	0.84 (0.76; 0.89)	4.14 (2.69; 6.38)	0.39 (0.31; 0.49)	10.64 (5.82; 19.46)
	MetaDTA	0.67 (0.61; 0.74)	0.84 (0.76; 0.89)	4.14 (2.70; 6.36)	0.39 (0.31; 0.49)	10.64 (5.83; 19.40)
Honest et al. 2002 [28]	Meta-DiSc 2.0	0.58 (0.45; 0.71)	0.84 (0.78; 0.88)	3.57 (2.75; 4.63)	0.50 (0.38; 0.67)	7.14 (4.59; 11.13)
	METANDI	0.58 (0.44; 0.71)	0.84 (0.78; 0.88)	3.57 (2.75; 4.64)	0.50 (0.37; 0.67)	7.15 (4.57; 11.19)
	METADAS	0.57 (0.43; 0.70)	0.84 (0.77; 0.88)	3.49 (2.67; 4.56)	0.51 (0.38; 0.68)	6.82 (4.34; 10.71)
	MetaDTA	0.58 (0.45; 0.71)	0.84 (0.78; 0.88)	3.57 (2.75; 4.63)	0.50 (0.38; 0.67)	7.14 (4.59; 11.13)
Verde 2010 [30]	Meta-DiSc 2.0	0.96 (0.95; 0.97)	0.95 (0.94; 0.97)	20.61 (14.65; 29.0)	0.05 (0.04; 0.06)	452.28 (296.93; 688.90)
	METANDI	0.96 (0.95; 0.97)	0.95 (0.93; 0.97)	20.59 (14.59; 29.06)	0.05 (0.04; 0.06)	451.76 (295.52; 690.60)
	METADAS	0.96 (0.95; 0.97)	0.95 (0.94; 0.97)	20.61 (14.64; 29.01)	0.05 (0.04; 0.06)	452.28 (296.78; 689.26)
	MetaDTA	0.96 (0.95; 0.97)	0.95 (0.94; 0.97)	20.61 (14.65; 29.0)	0.05 (0.04; 0.06)	452.28 (296.93; 688.90)
Plana 2018 [21]	Meta-DiSc 2.0	0.76 (0.70; 0.82)	1.00 (1.00; 1.00)	535.19 (281.45; 1017.67)	0.24 (0.18; 0.31)	2257.34 (1183.08; 4307.05)
	METANDI	0.76 (0.70; 0.82)	1.00 (1.00; 1.00)	535.27 (279.68; 1024.40)	0.24 (0.18; 0.31)	2254.82 (1174.19; 4329.97)
	METADAS	0.76 (0.70; 0.82)	1.00 (1.00; 1.00)	535.55 (280.27; 1023.35)	0.24 (0.18; 0.31)	2258.72 (1179.04; 4327.09)
	MetaDTA	0.76 (0.70; 0.82)	1.00 (1.00; 1.00)	535.19 (281.45; 1017.67)	0.24 (0.18; 0.31)	2257.34 (1183.08; 4307.05)

## Discussion

Our goal was to update a previous version of the MetaDiSc software [9]. After this update, we are confident that MetaDiSc 2.0 can be in the league of available DTA meta-analysis software. The application unifies the main standard routines for diagnostic accuracy meta-analysis and prevents reviewers from choosing among the variety of R packages available for this purpose, since not all of them have the currently recommended methods for DTA meta-analysis. Additionally, novel reviewers using MetaDiSc 2.0 could well avoid the steeped learning curve associated with using R. Another Shiny web application, MetaDTA [26], developed by the United Kingdom National Institute for Health Research (NIHR) Complex Review Support Unit in 2019, is available to conduct DTA meta-analyses. Meta-DiSc 2.0 has an advantage over the MetaDTA software because of its capacity to perform meta-regression analyses and calculate additional measures to quantify heterogeneity.

The app has several limitations. The meta-regression analysis implemented is based on the assumption of equal variances for the random effects of the logit sensitivities and the logit specificities of the compared subgroups. This assumption may be reasonable in many situations, although it may not be in some reviews. It is worth noting that the bivariate I-squared statistic depends on sample size. For this reason, the comparison of I-squared values among meta-analyses with a different number of studies and a different number of diseased and non-diseased participants is limited.

The app does not allow comparing the accuracy of two diagnostic tests, and the current version does not incorporate the risk of bias assessment using the QUADAS-2 tool [31].

The development of this web application was led by the Clinical Biostatistics Unit of the Ramón y Cajal Research Institute (IRYCIS), a unit that has broad experience in diagnostic test synthesis research focused on supporting informed decision-making in the healthcare area. This constitutes a collaborative project for knowledge transfer between IRYCIS and the Complutense University of Madrid and is supported by an intramural project funded by the Ramón y Cajal Research Institute ("*Rapid diagnostic reviews for decision-making in healthcare: analysis of critical points and software development*", 2018). This project has also been funded by Instituto de Salud Carlos III through the project "PI19/00481" (Co-funded by European Regional Development Fund/European Social Fund; “A way to make Europe”/"Investing in your future"). The Biomedical Research Networking Center in Epidemiology and Public Health (CIBERESP) funds the subscription to the shinyapps.io platform where the app is hosted.

## Conclusion

We developed an updated version of Meta-DiSc for performing diagnostic test accuracy meta-analyses. All computational algorithms have been validated by comparing different statistical tools and published meta-analyses.

## Data Availability

Meta-Disc 2.0 is a free web application and can be accessed at www.metadisc.es. R code and example data are available at https://metadisc.sourceforge.io.

## Abbreviations

app: Application
CCHD: Critical congenital heart defects
CI: Confidence interval
CIBERESP: Biomedical research networking center in epidemiology and public health
DOR: Diagnostic odds ratio
DTA: Diagnostic test accuracy
FN: False negatives
FP: False positives
ID: Identifier
IRYCIS: Ramón y Cajal research institute
LCI: Low confidence interval
LR: Likelihood ratios
MOR: Median odds ratios
NIHR: National institute for health research
sROC: Summary receiver operating characteristic
TN: True negatives
TP: True positives
UCI: Upper confidence interval
